# Round the Hospitals

**Published:** 1917-10-20

**Authors:** 


### ROUND THE HOSPITALS.

The Council of the College of Nursing have received with regret a letter from Miss L. V. Haughton resigning her seat on the Council, owing to the state of her health. Miss Haughton's withdrawal from the Council of the College of Nursing is a grievous loss indeed, for she it was who proved to be the very life and soul of its early initiation and development. Indeed, we fear her interest was, as it no doubt is, so great in the success of the College of Nursing, that it led her grievously to overtax her strength. Hence we fear the serious illness which has kept her a prisoner and sufferer for so many months. The reports at the end of last August were so encouraging that we, with the staff of Guy's Hospital, and her myriad of friends and well-wishers, conceived the hope that Miss Haughton's serious and untimely illness would terminate happily, and that she might be able to resume some of her invaluable work in association with the College of Nursing and other important matters very near her heart.

Few workers of similar age have ever won the affection and esteem of all with whom she had been associated to the extent Miss Haughton has enjoyed them throughout the whole of her short but most valuable career. In The Hospital of August 18 last, page 401, we tried to do a measure of justice to Miss Haughton's great talents and devoted hospital work. Indeed, the feeling of affection for her is so strong, as her manuscript book demonstrates, that the names in it include those of the present sisters and hospital and institution nurses, the administrative staffs, past and present League members, friends interested in the Nurses' League, members of the medical staff, and indeed all who have met Miss Haughton while serving as matron in some or all of the hospitals she has enriched by her presence and system, and especially those who have had the privilege to enjoy her friendship, her interest or advice. Miss Haughton belongs to a type of women workers who are rare and all too few. No words of ours can exaggerate the sense of loss which will be caused by her retirement from the College Council, nor express adequately the force and sincerity of the good wishes which will follow her in her retirement and cause many heartfelt prayers to be offered for her speedy recovery and restoration. Miss Haughton is comparatively young in years, and we earnestly hope that she may yet recover complete health and strength, that her mind and energies may continue to be devoted to the best interests of the profession which she loves, and to its development and extended usefulness, by the exercise of her many talents, powers of initiation, and administrative gifts.

The vacancy caused on the College Council by the retirement of Miss Haughton will be filled by Miss L. Clark, matron of the West Ham Union Infirmary, Whipps Cross, Leytonstone, who has been offered and has accepted the vacant seat. Miss Clark was trained at the London Hospital, and her subsequent career reflects the greatest credit upon those who trained her, for she has proved herself to be the possessor of a clear brain, fruitful in ideas. She, too, is a capable organiser and an administrator who has the courage to make momentous changes, when needed, in the system she has to administer. Miss Clark has had the enterprise and judgment to establish the office of sister-tutor in connection with the nurse-training school attached to the West Ham Union Infirmary, a step which cannot fail to make that institution much sought after and treasured by the best type of probationers, who have the wisdom to desire to secure a thorough, practical, and technical knowledge of their profession, under a matron whose justice and uplifting standards have already won for her a high reputation, which is extending in quality and fame as the months pass. The gifts Miss Clark has proved herself to possess belong to a type which should be found most essential and helpful in the building up and completion of an organisation, conceived upon the best lines, to make the College of Nursing the great and indispensable centre of nurse development and education. This should win for the College of Nursing the position of the leading and most authoritative centre of active development and perfected training for nurses, which the world contains.

---

As we go to press we have received a letter signed by the Honorary Secretaries and Secretary of the Royal British Nurses' Association, which will be found on page 54. In THE HOSPITAL of October 6, page 17, we published a resolution of the General Council of the Royal British Nurses' Association to the effect that it was then (September 27) decided *nem. con.* that it would not be in the interest of the R.B.N.A. to accept the alterations to the Draft Supplemental Charter suggested by the Privy Council. The letter published on page 54 raises no new point, nor one material to this position, seeing that at the meeting of the members of the R.B.N.A. held on January 18, 1917, Mrs. Latter, in seconding the resolution that the agreement for the amalgamation of the College of Nursing, Limited, with the Royal British Nurses' Association be confirmed, stated: "I would remind them [*i.e.*, the members present] that it is the College of Nursing which is coming to us—not we who are going to the College of Nursing." Mrs. Latter's statement was not challenged, Sir Arthur Stanley being present, and all the facts were therefore accepted. Mr. Paterson at the same meeting in effect said it would be impossible to secure a more representative meeting of the R.B.N.A. than that held on January 18, 1917, which adopted the resolution of amalgamation, as *The Nurses' Journal* reports, "without one member voting against it." Mr. Paterson declared: "At first I was strongly opposed to this amalgamation. Now, however, that the scheme has been modified and the wishes of your Council agreed to, I firmly believe it will be for the ultimate good of your [*i.e.*, the nursing] profession. The Council of the College have met all the wishes of the R.B.N.A. Council in the most friendly and conciliatory spirit. It is not too much to say that we have obtained all for which we asked." Mr. Paterson had already said: "If it be argued that the College of Nursing gains more than our Association I will not dispute it. The point to consider, the only point, is this: Is the amalgamation for the ultimate good of the nursing profession?" May we, therefore, hope, whatever the final issue, that the Councils of the R.B.N.A. and the College, at any rate, will insist upon the continuance and upholding of this "most friendly and conciliatory spirit"? We may add that the quotations are taken from the report of the Special General Meeting of the Royal British Nurses' Association on January 18, 1917, as published in the Association's official paper, *The Nurses' Journal*, dated February 1917, pages 12 to 20 inclusive.

ON the second anniversary (October 12) of the death of Nurse Edith Cavell, M. Gaston de Leval, whom Sir Arthur Stanley introduced as the gallant Belgian gentleman who, at great personal risk, did his utmost to save Nurse Cavell from the death the Germans had planned for her, told in London (for the first time in England) the methods and details of her trial and death. Had Miss Cavell been a Chinese, or even a French or Belgian woman, she would never have been shot, but the German hatred of the English was above everything, and they wanted an English victim. The defence afforded her was a mockery, her counsel not being allowed to speak to his client, to advise her, or to see any papers. The evidence against her, he had been given to understand and believed, the Germans gained principally through a somnambulist who talked in her sleep. The Germans mesmerised her, and so gained knowledge they would not otherwise have possessed. When explaining to Mr. Roosevelt that even according to German law the shooting of Miss Cavell was indefensible, Mr. Roosevelt exclaimed with vigour and emphasis, "Never mind German law; what of the laws of humanity?" Before Nurse Cavell died she wrote a cheque for her mother: "To die at seven in the morning, October 12, 1915. To my mother," and signed the cheque, "Edith Cavell. She was shot." It was not true that she was in a fainting condition when she met her doom. She was quite calm, and exclaimed, "I must pardon even my enemies. I know what I have done, and I must die without hatred; but I am happy to have died for my country." Just before the end she wrote in her Bible: "Died 7 o'clock, 12th October, 1915. With love to my mother. Edith Cavell." M. de Leval declared with force and truth that the tragedy would for ever be a stain on the German name, which nothing would be able to efface. It is well that this authentic account of the actual circumstances of Nurse Edith Cavell's execution should be known throughout the world, and we are indebted to M. de Leval for enabling us to publish it in these columns. Every nurse might properly cut it out, mount it, and have it framed to hang in her room always.

---

DR. DUDLEY A. SARGEANT, Physical Expert at Harvard University, the American correspondent of the *Daily Mail* reports, has promulgated a new theory regarding the fitness of women for war. Nine times out of ten, a woman, from the standpoint of physical endurance, should make as good a soldier as a man. Pound for pound the average normal woman in good health can endure more pain, discomfort and fatigue, and can expend more muscular energy than the average normal man of similar condition. A woman, of necessity, comes nearer the primitive type than man. She is biologically more of the barbarian, and she has therefore more physical endurance. She can undergo many strains that man cannot. Withstanding cold, or thirst, or hunger, or physical privation of any sort, a woman can outlast man. The question arises, Is this good or bad news for the sex, and how do the most thoughtful women regard it?

Under the name of "The White Cross," "an organisation," the *Times* states, "is now being established in America, its founder being the well-known educationist, Dr. Maria Montessori." It is designed "to treat the children of war, to gather up the new human generation, and to save it by a special method of education." Dr. Montessori's suggestion is to prepare teacher-nurses, and "to go to the assistance of these depressed and terrified children who are threatened with the perils of degeneration." The plan suggested is to start a free course to prepare volunteers to undertake the intellectual care of children. It will include first-aid, a knowledge of nervous diseases, diet for infants and children, isolation, special psychology, domestic science, agriculture, language, and a theoretical and practical course in the Montessori method as specially applied to these children. Dr. Montessori will give her services gratuitously to prepare the White Cross workers, with the assistance of medical specialists in nervous diseases.

Subsequently it is planned to send out working groups to France, Belgium, Serbia, Roumania, Russia, and other European countries, each consisting of twenty persons—*i.e.*, the director or head of the group, a secretary, two social workers, a special nurse, five teachers, and ten heads of families. The heads of families are to take care of the children out of school hours (evening and night), each head having twenty children installed in a small cottage, where she would act as a mother to the children. The teachers are to take care of the children during the day, and each teacher is to have a class of forty children. A total of 200 children would thus be provided for. A nurse would have charge of those who fell ill, and the heads of families would be responsible for the cottage, its upkeep, the wardrobe, and the cooking, in which they would be assisted by the children, who would be taught to make beds, wash dishes, comb their hair, and dress and undress themselves; the children to say their prayers night and morning, and be taught to give greetings and have instruction in social habits. The teachers would educate the children in Montessori methods, including physiological observations. They would measure the children and teach them agriculture and other manual work. It is estimated that in each cottage ten women of belligerent countries, widows, orphan girls, and others, could help the heads of the families in the work, and be taught the methods in about three months. In each class in six months, by assisting in the work, ten educated women of a higher class could learn and practise the method sufficiently to hold a class of their own. "Thus after six months the staff would be multiplied by ten, the addition being refugees, war widows, and others."

Finally, Mr. C. A. Bang, of 20 Bedford Street, London, W.C., honorary organiser, in a typed translation of Dr. Montessori's speech at the Women's Board of San Diego, California, from which the foregoing is mainly taken, claims: "A directoress and a social worker, with the help of the secretary [in the course of six months] will have made the necessary preparation to multiply the work of succour tenfold. Thus, after six months, we should have fifty teachers, one hundred heads of families, and about 2,000 children. The five original teachers and the ten heads of families would remain supervisors and would continue to help by their guidance the war women, who will simultaneously be made useful, consoled and curatively treated, and would so find an object and a mission in life." The first questions which arise to a practical mind are where and how and when "the depressed and terrified children" to the number of 2,000 and more are to be found in the belligerent countries where misfortune has befallen them, and where they are to be put into training. What are the sounds and signs which will enable the Director or the Secretary, or the Two Social Workers, or a Special Nurse, or each of the Five Teachers, or The Ten Heads of Families to recognise these children who are threatened with the peril of degeneration? This practical point must be faced and dealt with before the organisers will be justified in appealing for the "co-operation of all in England, who are interested in this work, to ensure its success."

It is pleasant to have an opportunity of recording the zeal of those matrons who make a practice of providing their institutions as far as possible with home-made jam and other food accessories. Miss S. A. Long, the matron of the Joint Isolation Hospital of the Richmond, Heston, and Isleworth Committee at Mogden, has made 455 lb. of jam, and successfully preserved ten dozen bottles of fruit. The fruit would have been preserved in much greater quantities had it been possible to obtain the necessary bottles. The matron has further made sufficient chutney and pickles to last all the winter. The matron picked all the soft fruit herself, and sent a quantity of the apples to the soldier patients at Percy House Hospital. In the course of our inspections we have often experienced surprise at the magnificent work done in these directions by matrons of large hospitals up and down the country. It would be very interesting, and most encouraging to others, as well as calculated to promote efficiency and saving in food provision during war-time, if those matrons, who make jam-making a practice, would send us a brief notice of the work done at their hospital or institution.

☛ It is our invariable practice to pay for all items of news which we may publish. The minimum payment is 5s. Paragraphs of about twenty lines in length—that is to say, of 150 words or less—are preferred. Contributions should be addressed to The Editor, The Hospital, 28 and 29 Southampton Street, Strand, London, W.C., and should reach him by the first post on Mondays.

For Matrons' and Sisters' Appointments, see page 60

